# Phage Resistance Is Associated with Decreased Virulence in KPC-Producing *Klebsiella pneumoniae* of the Clonal Group 258 Clade II Lineage

**DOI:** 10.3390/microorganisms9040762

**Published:** 2021-04-06

**Authors:** Lucia Henrici De Angelis, Noemi Poerio, Vincenzo Di Pilato, Federica De Santis, Alberto Antonelli, Maria Cristina Thaller, Maurizio Fraziano, Gian Maria Rossolini, Marco Maria D’Andrea

**Affiliations:** 1 Department of Medical Biotechnologies, University of Siena, Viale Mario Bracci, 16, 53100 Siena, Italy; lucia.henrici@gmail.com; 2 Department of Biology, University of Rome “Tor Vergata”, Via della Ricerca Scientifica snc, 00133 Rome, Italy; noemi.poerio@gmail.com (N.P.); federica.desa92@gmail.com (F.D.S.); mcthaller@gmail.com (M.C.T.); fraziano@bio.uniroma2.it (M.F.); 3 Department of Surgical Sciences and Integrated Diagnostics (DISC), University of Genoa, Via Benedetto XV, 6, 16126 Genoa, Italy; vincenzo.dipilato@unige.it; 4 Department of Experimental and Clinical Medicine, University of Florence, Largo Brambilla, 3, 50121 Florence, Italy; albertoanton88@gmail.com (A.A.); gianmaria.rossolini@unifi.it (G.M.R.); 5 Microbiology and Virology Unit, Florence Careggi University Hospital, Largo Brambilla, 3, 50121 Florence, Italy

**Keywords:** *Klebsiella pneumoniae*, *Klebsiella pneumoniae* carbapenemase KPC, Sequence type 258, phage, *Podoviridae*, virulence, capsule, polysaccharide, phage resistance mechanism

## Abstract

Phage therapy is now reconsidered with interest in the treatment of bacterial infections. A major piece of information for this application is the definition of the molecular targets exploited by phages to infect bacteria. Here, the genetic basis of resistance to the lytic phage φBO1E by its susceptible host *Klebsiella pneumoniae* KKBO-1 has been investigated. KKBO-1 phage-resistant mutants were obtained by infection at high multiplicity. One mutant, designated BO-FR-1, was selected for subsequent experiments, including virulence assessment in a *Galleria mellonella* infection model and characterization by whole-genome sequencing. Infection with BO-FR-1 was associated with a significantly lower mortality when compared to that of the parental strain. The BO-FR-1 genome differed from KKBO-1 by a single nonsense mutation into the *wbaP* gene, which encodes a glycosyltransferase involved in the first step of the biosynthesis of the capsular polysaccharide (CPS). Phage susceptibility was restored when BO-FR-1 was complemented with the constitutive *wbaP* gene. Our results demonstrated that φBO1E infects KKBO-1 targeting the bacterial CPS. Interestingly, BO-FR-1 was less virulent than the parental strain, suggesting that in the context of the interplay among phage, bacterial pathogen and host, the emergence of phage resistance may be beneficial for the host.

## 1. Introduction

Due to the emergence and broad dissemination of multidrug-resistant (MDR) pathogenic bacteria [1,2], antibiotic resistance was considered by the World Health Organization (WHO) as one of the three most important threats for public health of the 21st century [3]. Among MDR pathogens *Klebsiella pneumoniae* is one of the species of particular concern, given its propensity to acquire multiple antibiotic resistance genes, possibly becoming resistant to all the antimicrobials available for human therapy, along with its aptitude to persist and disseminate in nosocomial environments [4]. For these reasons, the WHO included *K. pneumoniae* in the critical priority list of resistant pathogens for which new antimicrobials are urgently needed [5].

The pandemic diffusion of MDR *K. pneumoniae* in clinical contexts, particularly of carbapenem-resistant strains, is largely attributable to the clonal spread of strains belonging to a limited number of Sequence Types (ST) and their derivatives, including ST258/512, ST101, ST11 and ST15 [4,6], although new STs such as ST307 and ST147 [7,8] are emerging. Isolates of ST258/512 represented the major clonal group of *K. pneumoniae* which contributed to the spread of the *K. pneumoniae* carbapenemase (KPC) genes. Nowadays, they are still at an endemic stage in many countries, including several areas in Europe, and account for the majority of KPC-producing *K. pneumoniae* clinical isolates [6]. Suitable therapeutic alternatives that can replace or complement classic approaches with antibiotics are therefore strongly desirable.

Bacteriophage therapy, i.e., the use of prokaryotic viruses to treat bacterial infections, is one of the most promising alternative options to fight the battle against MDR bacteria, even if there is the need to deeply characterize these viruses and the host response to them before their potential clinical use. As for conventional antibacterial agents, one of the problems with phage therapy is the possible development of resistance. It is, therefore, essential to understand more deeply the mechanisms underlying the resistance to bacteriophages in order to monitor and possibly prevent this phenomenon. A major piece of information required for the use of phages in therapy is the definition of the bacterial molecular targets exploited for the early stage of the infection cycle. Indeed, the characterization of receptors exploited by phages for infecting bacteria, and of their stability and conservation across different strains of a given bacterial species, can provide precious information relevant to phage-spectrum and risk of emergence of phage-resistance. For example, phage cocktails composed of viruses having different targets for bacterial infection can be rationally formulated, with the advantage of maintaining the same host specificity, but lowering the chances of selecting single phage-resistant variants. Furthermore, some phages use bacterial pathogenic factors for their infection, and it could be expected that mutations in these targets, while leading to resistance to phage infection, would attenuate bacterial pathogenicity. This would theoretically help the host immune system to clear the infecting pathogen [9]. For these reasons, the determination of the molecular targets exploited by phages for bacterial infection, and the mechanisms responsible for the onset of phage resistance phenomena, are primary information for the evaluation of phages for therapeutic applications.

In this work, the possible genetic modifications underlying the resistance to the phage φBO1E [10] by its susceptible host *K. pneumoniae* KKBO-1 and their role in virulence [11] have been studied. The KKBO-1 strain belongs to the KPC-producing *K. pneumoniae* clade II of the Clonal Group (CG) 258 (KPC-Kp of CG258-2) [12,13], whose members are linked to difficult to treat infections due to their extended MDR phenotype. φBO1E is the first characterized lytic phage highly specific against isolates of KPC-Kp of CG258-2 and could therefore represent a component of novel formulations for the treatment of infections caused by these pathogens.

## 2. Materials and Methods

### 2.1. Bacterial Strains and Growth Conditions

The *K. pneumoniae* clinical strain KKBO-1 [11] was used as the host for phage propagation and to obtain phage-resistant derivatives. Lysogeny Broth (LB) or Lysogeny Broth Agar (LBA) (Liofilchem srl, Roseto degli Abruzzi, Italy) were used to grow bacterial strains. Soft agar for double layer plating was composed of LB solidified with 0.7% agar (Liofilchem). SM buffer (10 mM Tris-HCl, pH 7.5; 100 mM NaCl; 10 mM MgSO_4_) was used for suspending and titrating bacteria and phages. Productions of φBO1E were obtained by the double-layer overlay technique. Briefly, an O/N culture of strain KKBO-1 was pelleted by centrifugation at 4500× *g* for 10 min and concentrated 1:10 in SM buffer. A 0.1 mL aliquot of bacteriophage preparation with a titer of 1 × 10^7^ PFU/mL, previously filtered through 0.22 μm filter (Millipore, Burlington, MA, USA), was added to 0.2 mL of the concentrated bacterial suspension. The mixture was added to 4.7 mL of molten soft-agar and poured onto LBA plates. Following O/N incubation at 37 °C, phages were recovered by adding 5 mL of SM buffer to each plate and incubating for 2 h at room temperature with gentle shaking. The soft-agar layer together with SM buffer was then collected and centrifuged at 4500× *g* for 10 min. The supernatant was finally collected, filtered through a 0.22 μm filter and stored at 4 °C. Phage titers were determined by mixing 0.1 mL of serial ten-fold dilutions of φBO1E preparations with 0.2 mL of a concentrated suspension of the KKBO-1 strain, prepared as for productions, and with 4.7 mL of molten soft-agar. The mixture was finally poured onto LBA plates, which were then incubated O/N at 37 °C. For the determination of bacterial growth curves, a single colony grown O/N on LBA plates has been inoculated in 5 mL LB and incubated O/N at 37 °C at 250 rpm. Subsequently, optical density (OD) at 600 nm was determined by an ONDA V-10 PLUS spectrophotometer (Giorgio Bormac s.r.l., Modena, Italy) and the inoculum used to prepare 50 mL LB at 0.05 OD_600_. Bacterial suspensions were finally incubated at 37 °C at 250 rpm, grown for 7 h determining OD_600_ every 1 h.

### 2.2. Antimicrobial Susceptibility Testing

Minimum inhibitory concentrations (MIC) of selected antibiotics were determined by broth microdilution using lyophilized custom plates (Micronaut-S MHK; Merlin Diagnostika GmbH, Berlin, Germany) according to manufacturer recommendations, except for fosfomycin, which was tested using a reference agar dilution method [14]. Results were interpreted according to the European Committee on Antimicrobial Susceptibility Testing (EUCAST) breakpoints [15].

### 2.3. Selection of Phage-Resistant Mutants and Assessment of Their Frequency and Stability

A single colony of KKBO-1 was grown O/N at 37 °C in LB. The bacterial suspension was then diluted 1:100 in 20 mL of fresh LB, and further incubated until an OD_600_ value of 0.6 was reached. Cultures were then infected with φBO1E to reach a Multiplicity of Infection (MOI) of 100, incubated for an additional 24 h, and finally plated on LBA. Nine randomly selected colonies were picked and analyzed by the spot test to confirm the phage resistance phenotype. Lysogeny was tested by a PCR assay using primers targeting the φBO1E depolymerase gene [10]. The rate of emergence of KKBO-1 mutants resistant to φBO1E infection was determined as previously described [16]. Briefly, a single colony of KKBO-1 from a fresh plate was inoculated in 5 mL of LB and grown O/N at 37 °C. An aliquot of this suspension was then used to obtain 20 mL of LB at an OD_600_ of 0.05, which were subsequently grown to 0.5 OD_600_. One ml of the inoculum was mixed with 300 μL of φBO1E to reach a MOI of 100, while a control culture was set up by adding 1 mL of bacterial culture to 300 μL of SM. Cultures were incubated at 37 °C for ten minutes, and then 100 μL of serial tenfold dilutions, ranging from 1 × 10^−1^ to 1 × 10^−7^, were plated on LBA plates. After O/N incubation at 37 °C, the rate of appearance of φBO1E-resistant mutants was determined by computing the ratio between phage-treated and phage-untreated bacterial suspensions. Possible phage-resistant mutants were verified by spot test assay. Experiments were performed in triplicate. One mutant, designated BO-FR-1, was selected for subsequent experiments. Stability of resistance of BO-FR-1 to φBO1E infection has been assessed as previously described, with minor modifications [17]. Briefly, a single colony of BO-FR-1, grown O/N on LBA, was inoculated in LB aerobically (10 mL of LB in a 100 mL flask, in an orbital shaker operating at 180 rpm) for approximately 50 generations by subculturing the strain (1:100 dilution) every 10–12 h for 4 days. The culture was finally plated to obtain isolated colonies, which were then checked for phage-resistance by spot-test using 5 μL of a φBO1E suspension at ≈10^8^ PFU/mL.

### 2.4. DNA Extraction and Genome Sequencing

Whole bacterial DNA from BO-FR-1 was extracted by the phenol-chloroform method [18]. Aliquots were resolved by agarose gel-electrophoresis (0.75% *w/v*), stained with ethidium bromide (0.5 mg/L) and visualized under UV light. DNA was quantified by using a Qubit fluorometer (Thermo Scientific, Waltham, MA, USA) and subjected to Next Generation Sequencing (NGS) using the Illumina HiSeq platform and a 2 × 250 paired ends approach at an external facility (Novogene Co., Ltd., Beijing, China).

### 2.5. Bioinformatics Analysis

Raw reads were assembled by using the SPAdes v 3.8.2 software [19]. Nodes from assemblies of the phage-resistant mutant were then compared with the genome of KKBO-1 (NCBI Bioprojects PRJNA 214751) by using a custom pipeline employing bwa v 0.7.17 [20] and samtools v 1.11 [21], by Snippy v 4.4.3 or by CSI Phylogeny v 1.4 [22] to find out Single Nucleotide Polymorphism (SNP) variants. ProgressiveMauve development snapshot 2015-02-26 [23] and parsnp v1.1.2 [24] were used to detect possible large genomic insertions/deletions or translocations. The obtained SNPs were considered reliable only when called by at least two different software. Genomic regions that following this analysis were found to be different between the mutant and its parental strain were further filtered out to remove results likely due to alignment or sequencing errors, using previously described criteria [11].

### 2.6. Virulence Assays and Statistical Analysis

Virulence of BO-FR-1 was evaluated in a *Galleria mellonella* infection model and compared with that of the parental strain by using bacterial loads of 10^7^ or 10^8^ CFU/mL, corresponding to 10^5^ or 10^6^ CFU/larva, respectively. Briefly, larvae of *G. mellonella* (Sa.gi.p, Ravenna, Italy) weighing approximately 450–600 mg, were surface-disinfected with a cotton swab dipped in 70% ethanol (Sigma-Aldrich, St. Louis, MI, USA) and injected with 10 μL inoculum of *K. pneumoniae* KKBO-1 or BO-FR-1 into the larval haemolymph behind the last proleg, by using a 30-gauge needle mounted on a 500 μL syringe equipped with a PB600-1 repeating dispenser (Hamilton, Reno, NV, USA). Bacterial suspensions were prepared in 10 mM phosphate-buffered saline (PBS) pH 6.5 and a total of 10 larvae was used for each condition. Negative control groups, injected with PBS buffer only or untreated, were also included. Larvae were placed into Petri dishes and incubated at 35 ± 2 °C in the dark, in a humidified atmosphere with food, and daily examined for pigmentation and mobility. Time of death was recorded at 24, 48 and 72 h. For each experiment, the injected inoculum was checked by plating serial dilutions and enumerating colonies after 12–16 h of incubation at 37 °C. Five independent experiments were performed for each condition. Data from independent experiments were pooled and differences in mortality were assessed by log-rank (Mantel–Cox) test. *p* values < 0.05 were considered significant. Statistical analyses were performed using the GraphPad Prism v 6.01 software (GraphPad Software, Inc., La Jolla, CA, USA).

### 2.7. Complementation Experiments

The wild-type *wbaP* gene together with flanking regions was amplified by PCR using primers wbaP_KKBO-1_AvaI and wbaP_KKBO-1_EcoRI (Table 1) and genomic DNA of strain KKBO-1 as a template. The obtained amplicon was cloned into the pGEM-T-Easy vector by using the pGEM^®^-T Easy Vector System (Promega, Madison, WI, USA) following manufacturer instructions to obtain pGEM-wbaP. Ligation was electroporated into *Escherichia coli* XL-1 blue^®^ and transformants selected on LBA plates supplemented with 100 mg/L of ampicillin. The *wbaP* gene was then subcloned into pACYC184 following digestion of pGEM-wbaP with AvaI and EcoRI (New England Biolabs, Ipswich, MA, USA) and ligation of the purified plasmid fragment containing the *wbaP* gene with pACYC184 restricted with the same enzymes to obtain pACYC-wbaP. The pACYC-wbaP plasmid was finally electroporated into *K. pneumoniae* BO-FR-1 electrocompetent cells and transformants selected on LBA plates containing tetracycline at 40 mg/L to obtain BO-FR-1 (pACYC-wbaP). A colony-PCR assay was performed to detect BO-FR-1 (pACYC-wbaP) transformants. Briefly, colonies from a LBA plate were suspended in 200 μL of molecular-grade water, boiled at 95 °C for 10 min, and pelleted at 12,000× *g* for 2 min at 4 °C. An aliquot of 2 μL of this lysate was used as a template for PCR assays by using the Seq_wbaP_fwd + pACYC184_ExtSeq_EcoRIrev or the pACYC184_ExtSeq_Ava + Seq_wbaP_rev primers, following the cycling conditions used for cloning. The authenticity of the cloned fragments in pGEM-wbaP and pACYC-wbaP was verified by Sanger sequencing on both strands at an external sequencing facility (Eurofins, Rome, Italy) by using primers reported in Table 1.

## 3. Results

### 3.1. Isolation of KKBO-1 Mutants Resistant to φBO1E Infection

Colonies of KKBO-1 derivatives insensitive to φBO1E by spot-test emerged after infection of liquid cultures with the phage at an MOI of 100. These derivatives showed a rough phenotype, in contrast with the mucoid appearance of the parental strain (Figure S1) and appeared at a frequency of 2 × 10^−6^. PCR tests with primers targeting the φBO1E genome were negative, ruling out the possibility of lysogeny and suggesting the obtainment of KKBO-1 mutants insensitive to φBO1E infection. One phage-resistant mutant (BO-FR-1) that was confirmed to be isogenic by NGS (see below) was selected for further investigations.

### 3.2. Phenotypic Characterization of BO-FR-1

Growth curves of BO-FR-1 were superimposable to those of the KKBO-1 parental strain, suggesting that phage resistance in this derivative was not associated with a growth fitness cost (Figure S2). Antimicrobial susceptibility testing revealed an identical susceptibility profile for all tested antibiotics, suggesting that resistance to the φBO1E phage in *K. pneumoniae* KKBO-1 does not affect antibiotic susceptibility (Table S1). In order to evaluate the stability of the phage-resistant phenotype exhibited by BO-FR-1, this strain was cultured for ≈50 generations, and then 20 randomly selected isolated colonies were tested by spot test using a φBO1E suspension. Results of this experiment showed that 100% of the tested cells were still resistant to φBO1E infection, suggesting that the phage-resistance phenotype was very stable.

### 3.3. Phage Resistance in BO-FR-1 Is Associated with a Non-Sense Mutation in the wbaP Gene

**Figure 1:**
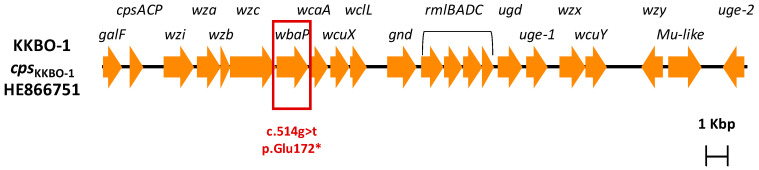
Genetic structure of the *cps* gene cluster of the KKBO-1 strain. The *wbaP* gene affected by the c.514g > t single nucleotide polymorphism leading to a premature termination (Glu172*) of the encoded glycosyltransferase is boxed.

Comparative analysis of the draft genomes of BO-FR-1 with the KKBO-1 parental strain excluded genomic rearrangements and revealed the presence of a single difference, i.e., a g→t transversion occurring at position 514 of the *wbaP* gene, resulting in a premature translation termination of the WbaP protein at position 172 (Figure 1). This gene, which is part of the capsular polysaccharide gene cluster of strains belonging to KPC-Kp of CG258-2, encodes the first glycosyltransferase necessary for the transfer of galactose-1-phosphate from UDP-galactose to the carrier lipid undecaprenyl phosphate [25,26].

**Figure 2:**
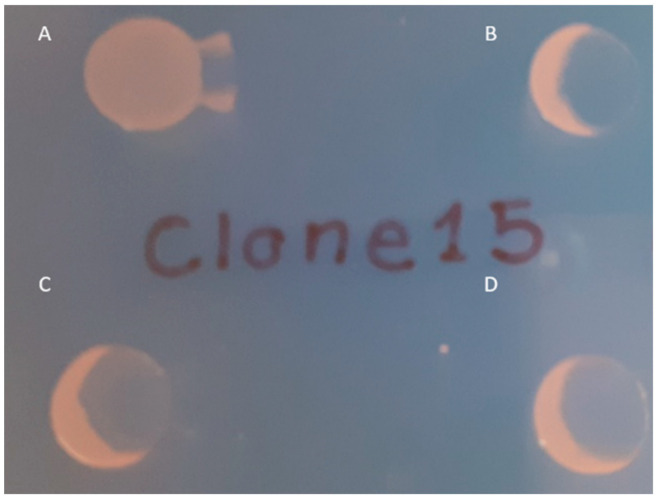
Spot test results confirming the restoration of susceptibility to φBO1E infection observed in BO-FR-1(pACYC-wbaP). Strains are as follows: BO-FR-1 (**A**), KKBO-1 (**B**), BO-FR-1(pACYC-wbaP) (**C**) and (**D**). Ten μL of cells in suspension of each strain were spotted on a LBA plate and partially layered with 5 μL of φBO1E (7 × 10^7^ PFU/mL).

To confirm the role of the mutated *wbaP* in conferring resistance to the phage φBO1E in BO-FR-1, a complementation experiment was performed using the pACYC-wbaP plasmid containing a copy of the constitutive *K. pneumoniae* KKBO-1 *wbaP* gene together with its own putative promoter. The introduction of the wild-type *wbaP* gene in BO-FR-1 was able to restore both the mucoid phenotype of this strain and its susceptibility to φBO1E infection (Figure 2 and Figure S1).

### 3.4. Virulence Comparison of KKBO-1 vs. BO-FR-1

**Figure 3:**
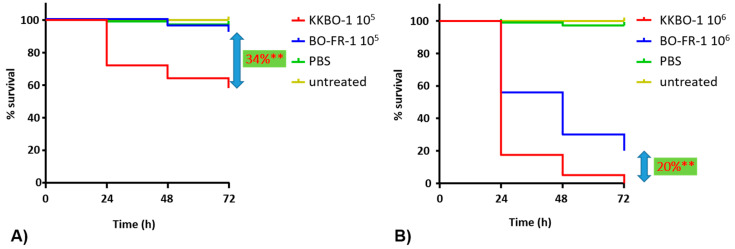
Kaplan–Meier survival curves of *Galleria mellonella* larvae. Infections have been performed with 10^5^ (**A**) or 10^6^ (**B**) cells of KKBO-1 or BO-FR-1. Data are the means of five independent experimental replicates. ** indicates *p* < 0.0001.

A *G. mellonella* infection model was employed to assess whether the mutation observed in BO-FR-1 resulted in changes in the virulence of KKBO-1. Results from these experiments showed a significant difference (*p* < 0.0001) in mortality rates of larvae when using either 10^5^ (34% decrease of mortality at 72 h) or 10^6^ (20% decrease of mortality at 72 h) bacterial cells for the infection (Figure 3). Collectively, these results demonstrated that a member of KPC-Kp of CG258-2 not expressing the capsular polysaccharide (CPS) exhibited a decreased virulence in this animal model, a result in line with the prominent role of CPS in the pathogenicity of several *Enterobacterales* species, including *K. pneumoniae*.

## 4. Discussion

In this work, the bacterial component exploited by φBO1E for the infection of KPC-Kp of CG258-2 has been characterized. A bioinformatic analysis suggested that the mutation observed in BO-FR-1 resulted in the potential production of a truncated WbaP protein lacking both the WcaJ (COG2148) and the bacterial sugar transferase domains (pfam02397), likely necessary for the correct physiological activity of WbaP and, ultimately, for the CPS production. This result was consistent with the observation of the rough phenotype displayed by BO-FR-1 and the other resistant derivatives and fits with the previously reported very narrow host range of this phage [10]. The φBO1E phage belongs to the *Podoviridae* family and is a member of the recently described *Drulisvirus* genus [27]. Even if the information on the infection specificity of *Drulisvirus* phages is currently available in a limited number of cases, i.e., KpV41, KpV48, KpV71, KpV74, KpV475 [28], myPSH1235 [29], πVLC1-4 [30] and vB_KpnP_IME337 [31], for most of these phages a perfect correlation between the bacterial *cps* type and phage susceptibility has been demonstrated [10,28,30,31] while this information is lacking or a broader specificity has been determined in a limited number of studies [32,33,34,35,36,37,38] (Table S2). This is in line with our results and, together with the observation that these phages possess a pectate lyase domain in their terminal fibers, suggests that this genus is characterized by the ability of targeting bacterial polysaccharides for the early infection steps.

The BO-FR-1 phage-resistant derivative exhibited no changes in the growth kinetics when compared to its parental strain, and its antimicrobial susceptibility appeared unaffected for all the tested antibiotics.

Susceptibility to the φBO1E phage was restored in the BO-FR-1(pACYC-wbaP) strain, although the phenotype of its colonies on LBA plates was not perfectly identical to that of the KKBO-1 strain (Figure S1). This suggests that BO-FR-1(pACYC-wbaP) expresses a different amount of CPS compared to KKBO-1, and this could be due to (i) the dysregulation of the *wbaP* gene expression which is not under its constitutive promoter in the complemented strain and/or to (ii) a polar effect resulting in the altered production of the glycosyl transferases present downstream *wbaP* in *cps*_KKBO-1_. However, *cps*_KKBO-1_ comprises a JUMP Start sequence upstream of the *wzi* gene, which is known to coordinate the transcription and expression of genes necessary for CPS synthesis [39,40]. This sequence contains the 5′-GGCGGTAG-3′ element able to upregulate transcription distal to a promoter suppressing polarity [41], thus supporting the hypothesis that the altered CPS production in BO-FR-1(pACYC-wbaP) is possibly due to the dysregulation of the *wbaP* gene expression when cloned in a medium copy number pACYC-derived plasmid.

Results of infections in the *G. mellonella* model revealed that the phage-resistant derivative appears less virulent compared to its parental strain, reinforcing the hypothesis that CPS is a major virulence factor of the *K. pneumoniae* species, especially for MDR clones such as those of ST258/512 in which other virulence factors are frequently lacking [4]. At the same time, our results demonstrate that CPS_KKBO-1_ constitutes a virulent factor *per se*, even if to a lesser extent than what has been observed with well-known CPS types associated with highly virulent strains, such as those of K1, K2 and K57. This information also suggests that φBO1E-resistant derivatives could be more sensitive to the host immune system, even if this hypothesis would deserve further investigation on other animal models. 

## 5. Conclusions

In conclusion, in this work, we have defined the bacterial component used by the *Podoviridae* φBO1E at its early stage of infection, also demonstrating that phage resistance is associated with the loss of a major virulent factor, i.e., the bacterial CPS. Our results, therefore, may suggest that during the course of an infection by KPC-Kp of CG258-2, which is eventually treated with φBO1E, the emergence of phage-resistant mutants could be beneficial for the host, which could be more able to clear the infection. Finally, the information derived from this work is of utmost importance for the rational design of phage cocktails to be used as additional tools for therapy. In this context, novel phage formulations composed of viruses with a very narrow host range, as in the case of φBO1E, may be part of novel “precision medicine” tools capable to specifically attack target bacteria with no or few effects on the resident, beneficial microbiota.

## Figures and Tables

**Table 1:** List of primers and PCR conditions used in this work. Endonuclease restriction sites added for cloning purposes in some primers are underlined.

Primer Name	Sequence (5′-3′)	Cycling Conditions (°C/min) ^a^	Purposes
wbaP_KKBO-1_AvaI	TAACGCTCGAGCGGTGTCCCAGTAAAAGG	D(94/1) A(52/1) E(72/1)	Cloning in pGEM-T-Easy^®^; Sequencing
wbaP_KKBO-1_EcoRI	AAGCAGAATTCACGCCAAATATCACCACCAT		Cloning in pGEM-T-Easy^®^; Sequencing
Seq_wbaP_fwd	GTGATGGCGGTGTTCCTG		Sequencing; Screening
Seq_wbaP_rev	GGTAGCCACGACAAATC		Sequencing; Screening
pACYC184_ExtSeq_Ava	GCTAACGGATTCACCACT		Sequencing; Screening
pACYC184_ExtSeq_EcoRIrev	CCTTTATTCACATTC		Sequencing; Screening

^a^ D stands for denaturation, A stands for annealing, and E stands for extension. All reactions include an initial denaturation step of 5 min at 94 °C and a final extension step of 5 min at 72 °C.

## Data Availability

Not applicable.

## Supplementary Materials

The following are available online at https://www.mdpi.com/article/10.3390/microorganisms9040762/s1, Figure S1: Appearance of colonies of the KKBO-1 and BO-FR-1 strains, Figure S2: growth curves of the KKBO-1 and BO-FR-1 strains, Table S1: antimicrobial susceptibility profiles of the KKBO-1 and BO-FR-1 strains, Table S2: host specificity of *Drulisvirus* phages.
