# Supplementary material for: Phage Resistance Is Associated with Decreased Virulence in KPC-Producing Klebsiella pneumoniae of the Clonal Group 258 Clade II Lineage
Source: Microorganisms. 2021 Apr 6;9(4):762. doi: 10.3390/microorganisms9040762 (PMC8067426; doi:10.3390/microorganisms9040762)
Supplement: Supplementary file 1 [file microorganisms-09-00762-s001.zip › microorganisms-1146918 supplementary to conversion/Fig S2.pdf]

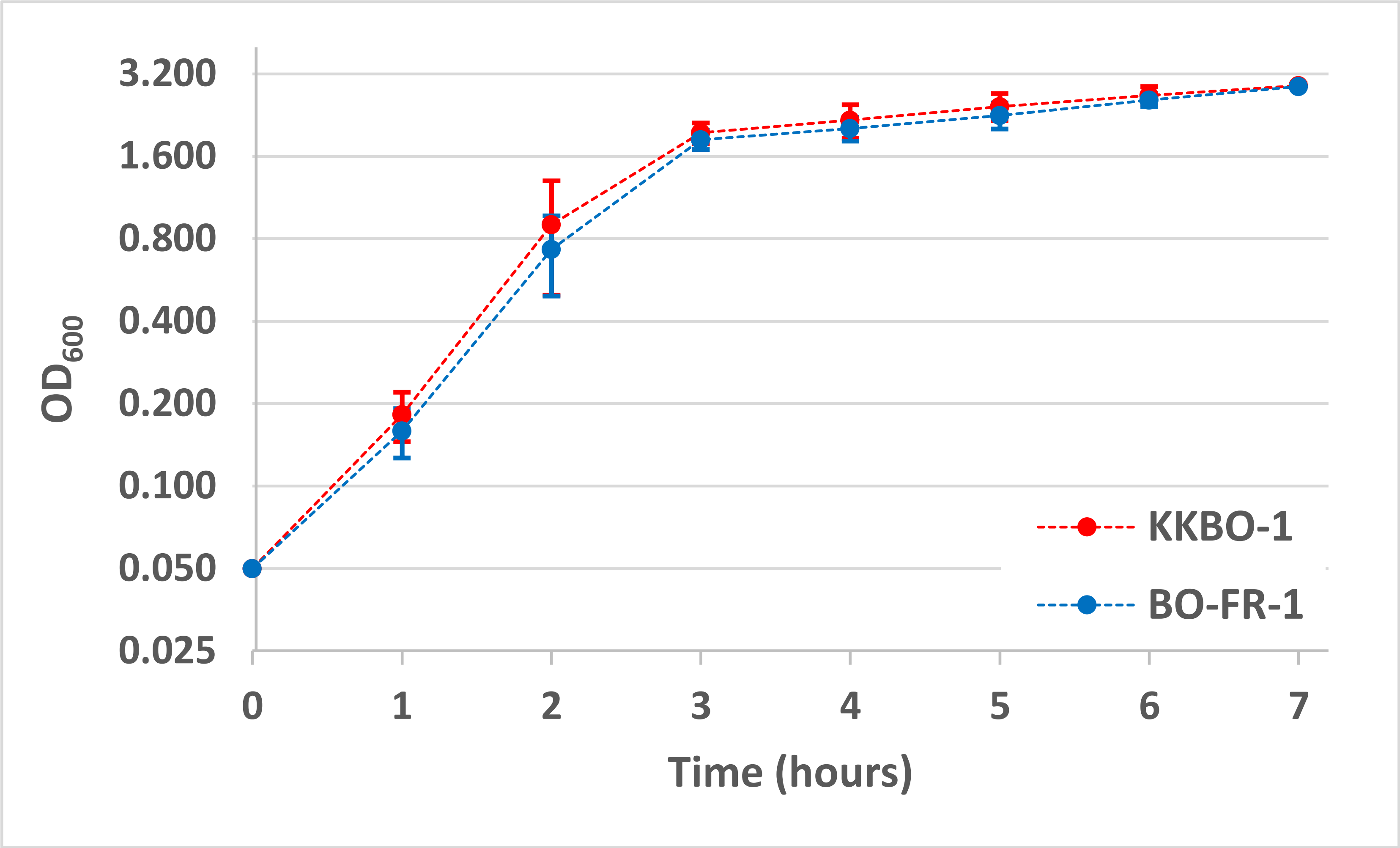

Figure S2. Growth curves of the phage-susceptible (red) and phage-resistant (blue) strains. Data are from the mean of three independent experiments. Vertical bars represent one standard deviation.
