# Supplementary material for: Phage Resistance Is Associated with Decreased Virulence in KPC-Producing Klebsiella pneumoniae of the Clonal Group 258 Clade II Lineage
Source: Microorganisms. 2021 Apr 6;9(4):762. doi: 10.3390/microorganisms9040762 (PMC8067426; doi:10.3390/microorganisms9040762)
Supplement: Supplementary file 1 [file microorganisms-09-00762-s001.zip › microorganisms-1146918 supplementary to conversion/Figure S1.pptx]

## Slide 1
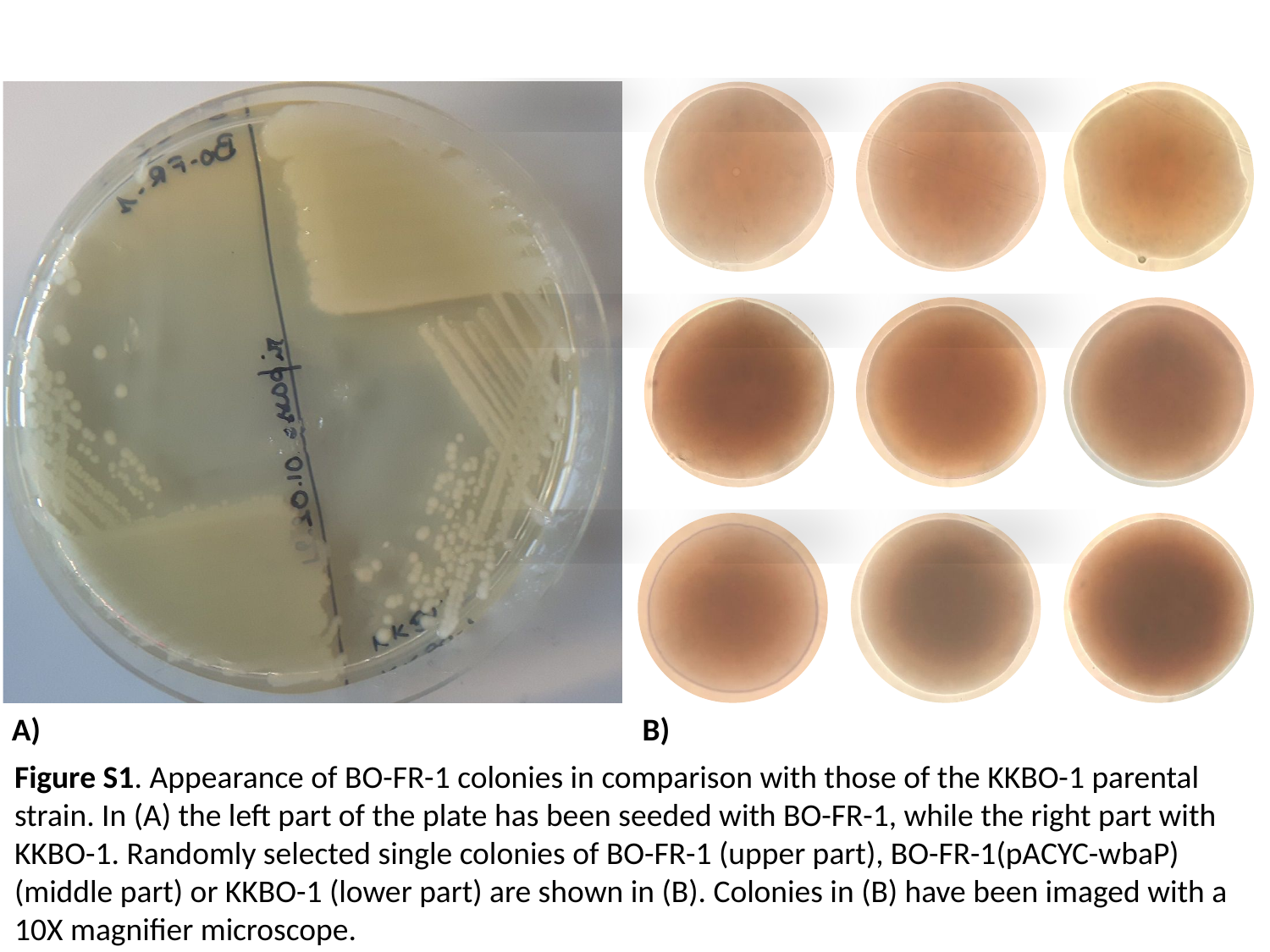

A)
B)
Figure S1. Appearance of BO-FR-1 colonies in comparison with those of the KKBO-1 parental strain. In (A) the left part of the plate has been seeded with BO-FR-1, while the right part with KKBO-1. Randomly selected single colonies of BO-FR-1 (upper part), BO-FR-1(pACYC-wbaP) (middle part) or KKBO-1 (lower part) are shown in (B). Colonies in (B) have been imaged with a 10X magnifier microscope.
