# Supplementary material for: Phage Resistance Is Associated with Decreased Virulence in KPC-Producing Klebsiella pneumoniae of the Clonal Group 258 Clade II Lineage
Source: Microorganisms. 2021 Apr 6;9(4):762. doi: 10.3390/microorganisms9040762 (PMC8067426; doi:10.3390/microorganisms9040762)
Supplement: Supplementary file 1 [file microorganisms-09-00762-s001.zip › microorganisms-1146918 supplementary to conversion/Table S1 and S2.pdf]

Table S1. Antibiotic susceptibility profiles of *K. pneumoniae* KKBO-1 and BO-FR-1. Assignments in the S or R categories for each antibiotic is reported near to MIC values. NC means not categorizable for the absence of clinical breakpoints.

Antibiotic	MIC (mg/l)	
	KKBO-1	BO-FR-1
Piperacillin/Tazobactam[a]	>128/4 (R)	>128/4 (R)
Amoxicillin/Clavulanate[b]	>64/2 (R)	>64/2 (R)
Cefepime	>16 (R)	>16 (R)
Ceftriaxone	>4 (R)	>4 (R)
Ceftazidime	>64 (R)	>64 (R)
Ceftazidime/Avibactam[c]	2/4 (S)	2/4 (S)
Ceftolozane/Tazobactam[a]	>64/4 (R)	>64/4 (R)
Ertapenem	>2 (R)	>2 (R)
Meropenem[1]	16 (R)	16 (R)
Tigecycline	0.5 (NC)	0.5 (NC)
Nitrofurantoin	>64 (NC)	>64 (NC)
Fosfomycin[2]	8 (S)	8 (S)
Trimethoprim/Sulfamethoxazole[d]	>8/152 (R)	>8/152 (R)
Amikacin	>16 (R)	>16 (R)
Gentamicin	≤1 (S)	≤1 (S)
Ciprofloxacin	>1 (R)	>1 (R)
Levofloxacin	>8 (R)	>8 (R)
Colistin	1 (S)	1 (S)

[a] Tazobactam at fixed concentration of 4 mg/l
[b] Clavulanate at fixed concentration of 2 mg/l
[c] Avibactam at fixed concentration of 4 mg/l
[d] Trimethoprim/sulfamethoxazole at 1:19 proportion

[1] Breakpoints for indications other than meningitis have been applied
[2] Breakpoints for intravenous administration have been applied

Table S2. Host specificity of phages belonging to the *Drulisvirus* genus reported by the International Committee on Taxonomy of Viruses (last accessed on 18/02/2021).

Host (Genus)	Phage Name	Reported Host-Specificity	Reference
Escherichia	Minorna	-	[32]
Klebsiella	AltoGao	-	[33]
Klebsiella	φBO1E	$CPS_{KKBO\text{-}1}$	[10]
Klebsiella	F19	-	-
Klebsiella	K244	-	-
Klebsiella	Kp2	-	-
Klebsiella	KP34	-	[34]
Klebsiella	KP-Rio/2015	-	[35]
Klebsiella	KpS2	-	[36]
Klebsiella	KpV41	CPS_{K1}	[28]
Klebsiella	KpV48	broad	[28]
Klebsiella	KpV71	CPS_{K1}	[28]
Klebsiella	KpV74	CPS_{K2}	[28]
Klebsiella	KpV475	CPS_{K1}	[28]
Klebsiella	KPV811	-	[37]
Klebsiella	myPSH1235	broad	[29]
Klebsiella	SU503	-	[38]
Klebsiella	SU552A	-	[38]
Shigella	SFN6B	-	-
Klebsiella	πVLC1-4[1]	CPS_{K22}	[30]
Klebsiella	vB_KpnP_IME337[1]	CPS_{KN2}	[31]

[1]Not yet included in the ICTV list.
